# Clinical Experience of Intense Pulse Light Therapy Along With Meibomian Gland Expression in Patients With Meibomian Gland Dysfunction: A Retrospective Study

**DOI:** 10.7759/cureus.105843

**Published:** 2026-03-25

**Authors:** Vijay Shetty, Nitin S Deshpande, Prajakta Deshpande, Jekin Choubisa, Akshay Chavan, Seeba John, Alfin Shaji, Amruta Pradhan, Ojas N Deshpande, Anjali Sharma, Maninder S Setia

**Affiliations:** 1 Ophthalmology, Shree Ramkrishna Netralaya, Thane, IND; 2 Optometry, Shree Ramkrishna Netralaya, Thane, IND; 3 Clinical Research, Shree Ramkrishna Netralaya, Thane, IND; 4 Epidemiology, Shree Ramkrishna Netralaya, Thane, IND

**Keywords:** intense pulsed light, lipid layer thickness, longitudinal study, meibomian gland dysfunction, osdi scores, side effects

## Abstract

Introduction: Intense pulsed light (IPL) therapy alone or along with meibomian gland expression (MGX) is one of the therapies for the management of dry eye symptoms associated with meibomian gland dysfunction (MGD). The present study was conducted to assess changes in dryness of the eyes, as assessed by ocular surface disease index (OSDI) scores, and lipid layer thickness in patients with MGD treated with IPL along with MGX. We also evaluated the side effects of IPL treatment in these patients.

Methods: This study was a retrospective secondary longitudinal data analysis of 200 eyes of 100 patients with MGD and symptoms of dry eye. They were treated with three sessions of IPL along with MGX at a tertiary eye care center in India. We abstracted data from 100 patients who presented to the clinic, were clinically diagnosed with MGD, underwent three sessions of IPL therapy (with a 15-day gap between sessions), and were followed up for evaluation 30 days after the last treatment. The following parameters were included in data abstraction: demographics (age, gender) and clinical parameters (complaints, symptoms, comorbidities, current treatment). The main outcomes were as follows: OSDI score and change in OSDI scores; lipid layer thickness category at each visit and change in lipid layer thickness; and side effects after IPL sessions. Multiple eyes from the same patient were accounted for using mixed-effects models for multivariate analysis.

Results: The mean (SD) age of the patients was 65.2 (9.7) years, with 52 females and 48 males. At baseline, the proportion of eyes with a lipid layer thickness value of ≤15 nm was 24% (n = 48), a value of 30 nm was 45% (n = 90), a value of 30-80 nm was 19% (n = 38), and a value of ≥80 nm was 12% (n = 24). There was an improvement in lipid layer thickness at each visit, and by the last follow-up, the proportions had changed to 23% (n = 46) for ≤15 nm, 33.5% (n = 67) for 30 nm, 18.5% (n = 37) for 30-80 nm, and 25.0% (n = 50) for ≥80 nm; this change was statistically significant (p < 0.01). The mean ± SD OSDI score at baseline was 14.62 ± 9.73. It reduced to 11.07 ± 8.41 on day 15 and was 7.35 ± 6.80 at the last follow-up. The mean change from baseline to day 60 was −7.27 ± 7.19, which was statistically significant (p < 0.001). In multivariate mixed-effects models, we found a significant reduction in the OSDI score at every follow-up visit (estimate: −2.34, 95% CI: −2.60 to −2.07, p < 0.001). None of the patients reported any side effects.

Conclusions: There was a reduction in OSDI scores at the last follow-up visit compared with the values before the first session of IPL in this retrospective study. We also observed a significant reduction in OSDI scores after each session of IPL and MGX. Moreover, there was a significant increase in the proportion of eyes with higher lipid layer thickness at the final follow-up visit, indicating improvement in lipid layer thickness. The IPL sessions were well tolerated, with no reported side effects.

## Introduction

Meibomian gland dysfunction (MGD) is associated with reduced meibomian gland density or lipid secretion; this may lead to destabilization of the tear film, ocular inflammation, and symptoms of dry eye disease [1,2]. The International Workshop on Meibomian Gland Dysfunction has defined it as "Meibomian gland dysfunction (MGD) is a chronic, diffuse abnormality of the meibomian glands, commonly characterized by terminal duct obstruction and/or qualitative/quantitative changes in the glandular secretion. It may result in alteration of the tear film, symptoms of eye irritation, clinically apparent inflammation, and ocular surface disease" (Nichols et al, page 1922, Investigative Ophthalmology & Visual Science, 2011) [3]. The global prevalence of MGD is approximately 35.8%, and it may be as high as 83% in some populations [4-6]. Although some authors have found a higher prevalence in males compared with females, others have found no significant difference between these genders in clinical studies, whereas a few have shown a higher prevalence in females [4,6,7]. Various factors such as age, systemic conditions, ocular graft versus host disease, medications, and excessive digital display may be associated with MGD [8-14].

Multiple treatment modalities have been recommended for the management of MGD. Some of these include procedures that patients can perform at home, such as eyelid hygiene, warm compresses, and warming eye masks [2,14-17]. However, compliance is an important issue in the proper implementation of these strategies [18,19]. Other treatment options include lubricating eye drops and topical and oral antibiotics [15,20-22]. Recently, there have been many studies assessing the role of topical cyclosporine A (calcineurin inhibitor), lifitegrast (lymphocyte function-associated antigen-1 antagonist), loteprednol etabonate (corticosteroid), or diquafosol solution (P2Y2 agonist) in MGD, and these medications have also been found to be useful in MGD [23-27]. However, the outcome of management of MGD may be dependent on patient compliance. Some in-office procedures, such as manual expression of meibomian glands, microblepharoexfoliation, intraductal probing, and other procedures, such as thermal pulsation and intense pulsed light (IPL) therapy, have also been found to be useful in the management of MGD [28-33]. Many observational studies and trials have assessed the outcomes of IPL in patients with MGD, and they have reported improvement in inflammatory markers, tear breakup time, dryness parameters, and meibomian gland parameters [33-36]. The potential mechanisms of action of IPL include its anti-inflammatory, antimicrobial, and antioxidative effects, photomodulation, downregulation of epithelial turnover, destruction of superficial blood vessels, and meibum fluidification and alteration of lipids [34,37-49]. Some side effects reported were light sensitivity, redness, edema, blistering, and mild pain and burning sensation [34,50-53].

Previous reviews have also provided evidence on the role of IPL in MGD. Miao and colleagues found that IPL alone or in combination with meibomian gland expression may be useful in improving symptoms in patients with MGD [54]. Leng and coworkers also found that IPL combined with meibomian gland expression is safe and effective in the management of MGD. However, they also reported that IPL alone may not be superior to meibomian gland expression in these patients [55]. Thus, potentially, IPL may be effective in the reduction of symptoms in these patients. The present study was conducted to evaluate changes in dryness of the eyes (as assessed by ocular surface disease index scores) and lipid layer thickness (both these outcomes as measures of effectiveness) in patients with MGD treated with three sessions of IPL, along with meibomian gland expression over an observation period of 60 days. We also assessed the side effects of IPL treatment in these patients during this time.

## Materials and methods

Study design

This study was a retrospective secondary longitudinal data analysis of 200 eyes of 100 patients with MGD and symptoms of dry eye. They were treated with three sessions of IPL along with meibomian gland expression (MGX).

Study site and population

The study was conducted at Shree Ramkrishna Netralaya Super Speciality Eye Care, India. This is a tertiary eye care center with multiple eye specialties such as cataract, glaucoma, and vitreoretinal disorders. We abstracted data from 100 patients who presented to the clinic and were clinically diagnosed with MGD. These patients were started on lubricants and had complaints and symptoms of dry eye associated with MGD, and the clinician suggested additional management or escalation of treatment. We included data from patients who underwent three sessions of IPL therapy and were followed up for evaluation 30 days after the last treatment. We did not include data from patients who had received IPL or other light-based therapy in the past one year. The total observation time was 60 days (±7 days), which was the last follow-up visit.

Procedure and follow-up

We use the ThermaEye® Plus (MDS Medical Technologies S.L., Barcelona, Spain) IPL laser in these patients. We placed a metal ocular shield on the patients’ eyes and applied cooling gel over the periocular area along the lower eyelid, bilaterally extending from the medial to the lateral canthus. We used the 650-nm filter with the handpiece and set the fluence at 8.0 J/cm^2^. We delivered two flashes per eye during each cycle, one flash over the temporal (lateral canthal) periocular region and the other on the lower eyelid region. Each flash delivered two consecutive pulses with a pulse width of 3 ms, a flash duration of approximately 20 ms, and a repetition interval of 2.0 seconds between successive flashes. The energy emission was controlled by the handpiece finger switch. The entire sequence was repeated two times for each eye. After the procedure, the patients were instructed to use topical sunscreen for the next three days whenever they went outdoors and to wash their faces with lukewarm water. All patients received three sessions of IPL. The IPL sessions were followed by meibomian gland expression by the clinician. The patients continued the use of lubricant eye drops.

Study outcomes and variables

All data were abstracted from hospital medical records. The following parameters were included in data abstraction: demographics (age, gender) and clinical parameters (complaints, symptoms, comorbidities, current treatment). Besides these parameters, we also abstracted data from the LacryDiag (Quantel Medical, USA) measurements. These included quantitative analysis of meibomian glands, including percentage loss in the upper and lower eyelids, and grading of lipid layer thickness. The percentage loss of meibomian glands for each eyelid was classified and scored as follows: None, 0; <33%, 1; 33-66%, 2; >66%, 3, individually for the upper and lower lid [56]. A total score for total meibomian gland loss was calculated by adding the individual scores for each eyelid (example, if the score for the right eyelid was 2 and for the left eyelid was 1, then the total score was 3) [56,57]. The lipid layer thickness is shown on a graded scale, and the color of the thickness is based on this graded scale: <15 nm, 15 nm, 30 nm, 30 to 80 nm, 80 nm, 80 to 120 nm, 120 to 160 nm. We divided them into four groups (≤15 nm, 30 nm, 30 to 80 nm, and ≥80 nm). However, we also compared the change in the lipid layer thickness based on the original categories. If the category increased at subsequent follow-up, that is, from <15 nm to 15 nm, it was considered “improvement”; if the category decreased, that is, from 15 nm to <15 nm, it was considered “worsening”; and if the category remained the same, it was considered “no change.” We also abstracted ocular surface disease index scores (OSDI) (Antares+ Corneal topographer, Costruzione Strumenti Oftalmici, Firenze, Italy). The OSDI scores were classified into four categories: Normal: <13, Mild: 13-22, Moderate: 23-32, and Severe: ≥33 [58-62].

The visits were as follows: first visit (baseline visit, day 0), clinical evaluation, lipid layer analysis, OSDI assessment, and first session of IPL; second visit at day 15 (±3 days), clinical evaluation (including history of any side effects), lipid layer analysis, OSDI assessment, and second session of IPL; third visit at day 30 (±5 days), clinical evaluation (including history of any side effects), lipid layer analysis, OSDI assessment, and third session of IPL; and fourth visit at day 60 (±7 days).

Outcomes

The main outcomes were as follows: OSDI score and change in OSDI scores; lipid layer thickness category and change in lipid layer thickness; and side effects after IPL sessions.

Statistical methods

Data were entered in MS Excel (Microsoft Corporation, Redmond, Washington) and converted to Stata Version 17 (StataCorp, College Station, Texas) for analysis. We assessed the normality of linear data using the Shapiro-Wilk test. The linear data were described as mean and standard deviation for normal or median and interquartile range (IQR) for non-normal data. The categorical data were described as proportions. The values across multiple visits were compared using the Friedman rank sum test, followed by post hoc pairwise comparison with Bonferroni correction for non-normal data. We used the Kruskal-Wallis test followed by Dunn’s test with Bonferroni correction for comparison of non-normal data across more than two groups. We used the McNemar-Bowker test and the Stuart-Maxwell test for marginal homogeneity for paired categorical data. Since we had multiple eyes from the same patient, we used mixed-effects models for multivariate analysis [63,64]. The fit of the model was assessed using the Akaike Information Criteria and Bayesian Information Criteria [65-67]. The outcome was a change in the OSDI score, and the variables in the model were age, gender, comorbidities, duration, and severity of meibomian gland loss at baseline. A p-value of <0.05 was considered statistically significant.

Ethics approval

The study was approved by the Ethics Committee of Shree Ramkrishna Netralaya Super Specialty Eye Clinic (Reference Number: SRNEC/ECD/2025/015, dated August 20, 2025). Since this was a retrospective study of secondary data, a waiver of consent was requested and granted by the Ethics Committee. All data were anonymized, and no identifiers were included in the dataset.

## Results

We present results from 200 eyes of 100 patients who underwent IPL for MGD. The mean ± SD age was 65.2 ± 9.7 years, and the gender distribution was 52 females and 48 males. The following comorbidities were recorded in these eyes: hypertension (50%, n = 50), diabetes mellitus (25%, n = 25), glaucoma (9%, n = 9), and lipid disorders (10%, n = 10). All eyes included in this analysis had meibomitis, and the other common symptoms were foreign body sensation (118, 59%), itching (116, 58%), and tiredness of eyes (94, 47%). Details have been presented in Table 1. At baseline, both eyelids mostly had none to <33% meibomian gland loss (Table 1). The median duration to the first IPL session after diagnosis of MGD at the clinic was 11 days (IQR: 6 to 18.5 days).

**Table 1:** Table showing the demographic and clinical characteristics of 200 eyes from 100 patients at baseline, Maharashtra, India The denominator for demographics (age, gender) and comorbidities was 100 (number of patients), and for others, it was 200 (number of eyes).

Parameter	Estimate
Age (years)	
Mean ± SD	65.2 ± 9.7
Gender, n (%)	
Female	52 (52.0)
Male	48 (48.0)
Co-morbidities, n (%)	
Glaucoma	9 (9.0)
Diabetes mellitus	25 (25.0)
Hypertension	50 (50.0)
Lipid disorders	10 (10.0)
Other	11 (11.0)
Clinical features/symptoms, n (%)	
Redness	13 (6.5)
Foreign body session	118 (59.0)
Itching	116 (58.0)
Tiredness of the eyes	94 (47.0)
Anterior blepharitis	22 (11.0)
Meibomitis	200 (100.0)
Meibomian gland loss, n (%)	
Upper lid	
None	39 (19.5)
<33%	141 (70.5)
33-66%	17 (8.5)
>66%	3 (1.5)
Lower lid	
None	39 (19.5)
<33%	137 (68.5)
33-66%	19 (9.5)
>66%	5 (2.5)
Total lid severity score [N (%)]	
0	6 (3.0)
1	57 (28.5)
2	105 (52.5)
3	24 (12.0)
4	5 (2.5)
5	3 (1.5)
Time between diagnosis and first IPL session (days)	
Median (IQR)	11 (6, 18.5)
Time categories, n (%)	
≤7 days	78 (39.0)
8-15 days	60 (30.0)
≥16 days	62 (31.0)

**Figure 1:**
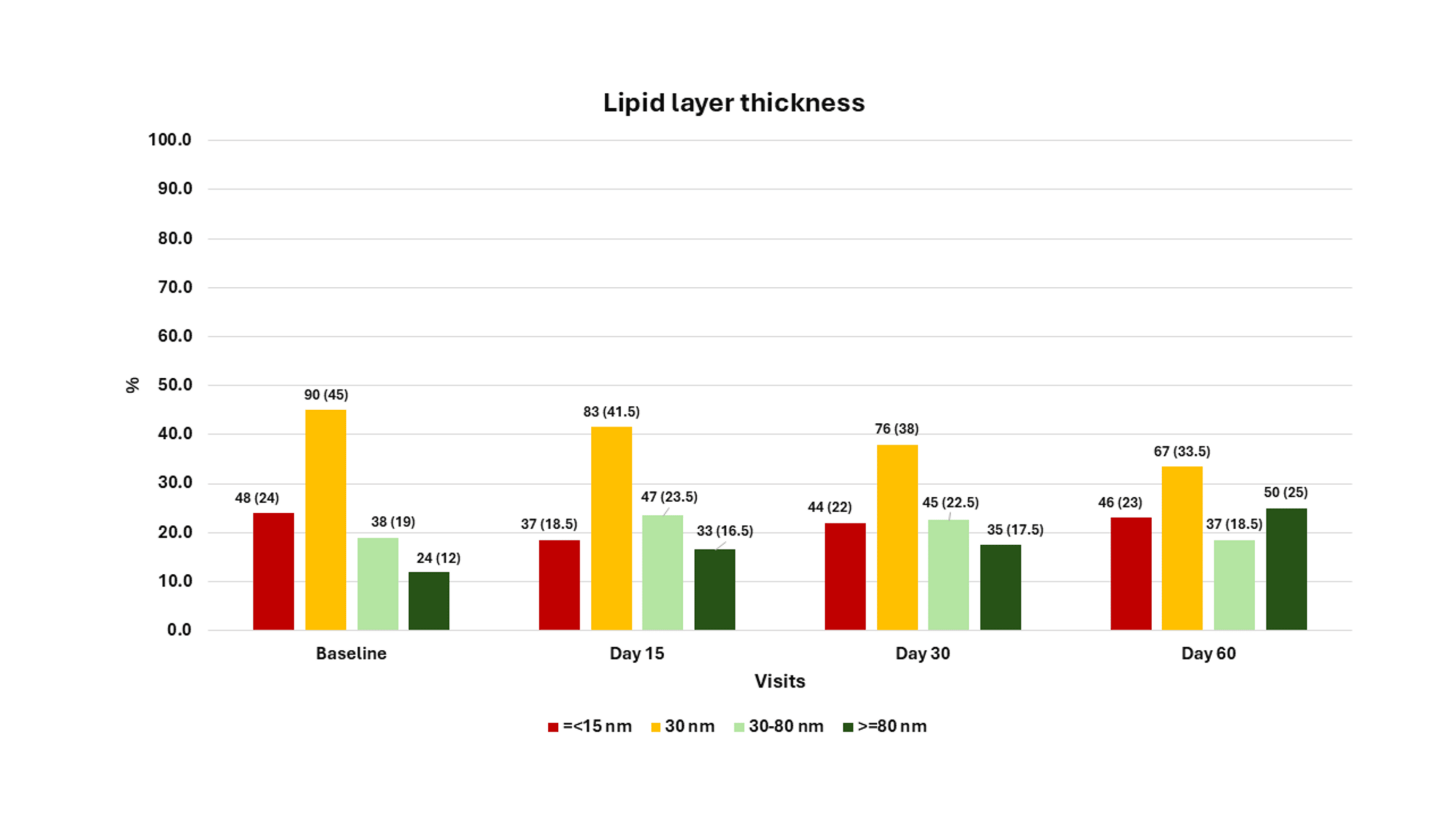
The categories of lipid layer thickness at different visits in 200 eyes from 100 patients, Maharashtra, India The x-axis shows the visit, and the y-axis shows the proportion of cases. The values are n (%) for the bars.

**Figure 2:**
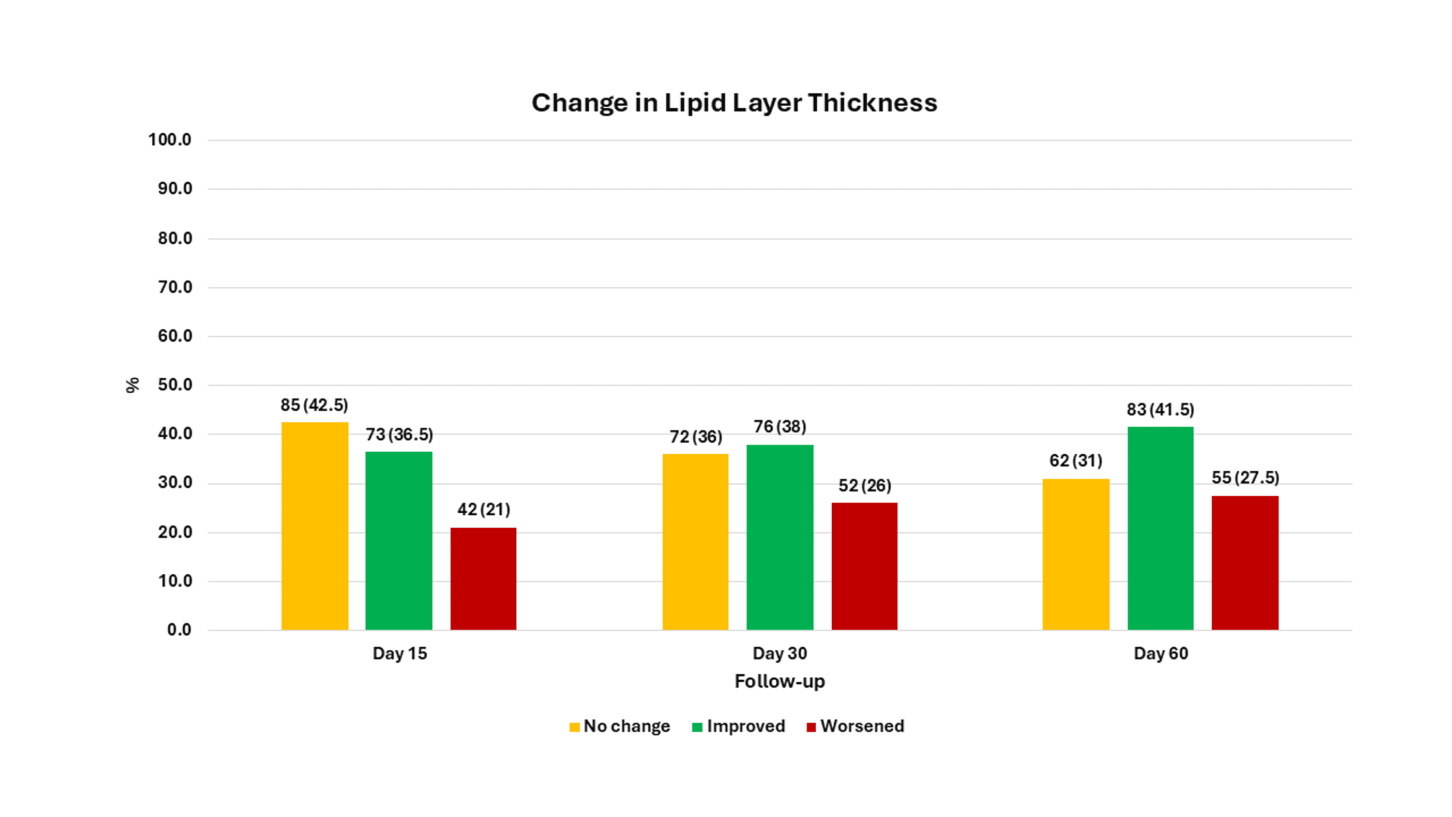
The changes in lipid layer thickness categories at follow-up visits in 200 eyes from 100 patients, Maharashtra, India The x-axis represents the follow-up visit, and the y-axis represents the proportion of cases. The values are n (%) for the bars.

At baseline, the proportion of eyes with a lipid layer thickness value of ≤15 nm was 24% (n = 48), a value of 30 nm was 45% (n = 90), a value of 30-80 nm was 19% (n = 38), and a value of ≥80 nm was 12% (n = 24). There was an improvement in lipid layer thickness at each visit, and by the last follow-up, the proportions had changed to 23% (n = 46) for ≤15 nm, 33.5% (n = 67) for 30 nm, 18.5% (n = 37) for 30-80 nm, and 25.0% (n = 50) for ≥80 nm; this change was statistically significant (p < 0.01) (Figure 1). Upon evaluating changes in lipid layer thickness categories, we found that 36.5% (n = 73) of eyes improved, 42.5% (n = 85) showed no change, and 21.0% (n = 42) worsened on the second visit compared with baseline. By the last visit, 41.5% (n = 83) showed improvement, 31.0% (n = 62) showed no change, and 27.5% (n = 55) showed worsening of lipid layer thickness compared with baseline (Figure 2). There was no significant association between the severity of meibomian gland loss at baseline and changes in lipid layer thickness at each follow-up visit. Furthermore, there was no association between the time to IPL after diagnosis of MGD and changes in lipid layer thickness at the follow-up visits.

**Figure 3:**
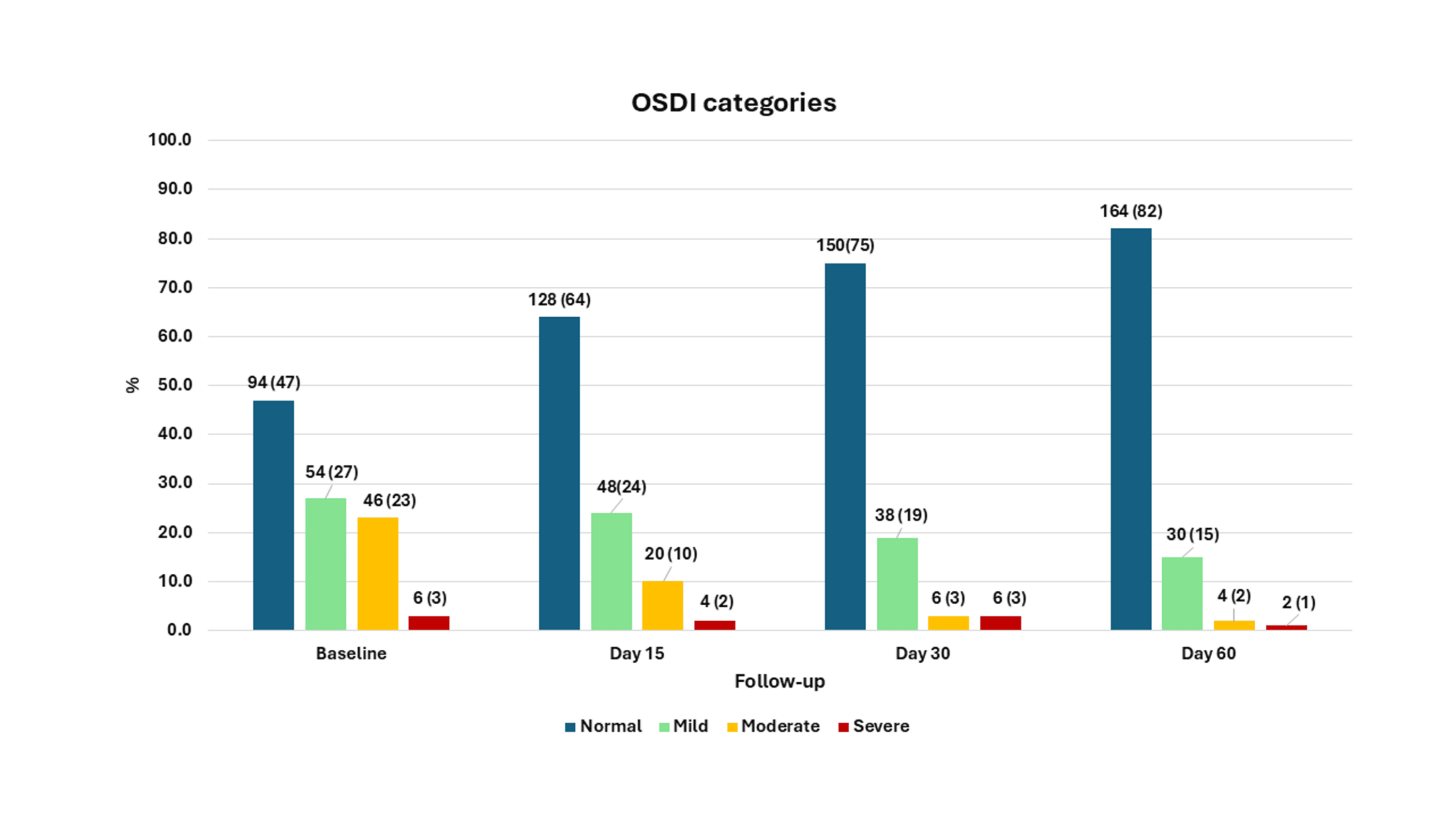
Figure showing the OSDI categories at different visits in 200 eyes from 100 patients, Maharashtra, India OSDI: Ocular Surface Disease Index scores. The x-axis represents the visits, and the y-axis represents the proportion of eyes. The values are n (%) for the bars.

The mean ± SD OSDI score at baseline was 14.62 ± 9.73. It reduced to 11.07 ± 8.41 on day 15 and was 7.35 ± 6.80 at the last follow-up. The mean change from baseline to day 60 was −7.27 ± 7.19, which was statistically significant (p < 0.001) (Table 2). Thus, 94 (47%) of eyes were classified as normal on OSDI, 54 (27%) as mild, 46 (23%) as moderate, and 6 (3.0%) as severe. The proportion of eyes classified as normal increased at every visit, and at the last follow-up, this was as follows: normal 164 (82%), mild 30 (15%), moderate 4 (2%), and severe 2 (1%); this difference was statistically significant (p < 0.001) (Figure 3). There was no significant association between the severity of meibomian gland loss and changes in OSDI scores at each follow-up visit. At the first follow-up visit, although the reduction in OSDI scores was higher in those in whom the first IPL session was started within the first seven days of diagnosis compared with 15 days later, the difference was not statistically significant. At the last follow-up visit, the change was nearly similar across all groups (Table 3). We compared the mean OSDI score according to time of start at baseline and did not find any statistically significant difference across time categories (≤7 days, 8 to 15 days, ≥16 days).

**Table 2:** The ocular surface disease index scores (OSDI) at multiple visits and changes in OSDI scores in 200 eyes from 100 patients, Maharashtra, India We used the Friedman rank sum test followed by post hoc pairwise comparisons with Bonferroni correction for non-normal data. The Friedman test statistic was 224.07 (chi-square, degrees of freedom = 3), with an overall p-value < 0.0001. Baseline vs day 15: p < 0.001; baseline vs day 30: p < 0.001; baseline vs day 60: p < 0.001; day 15 vs day 30: p = 0.21; day 15 vs day 60: p < 0.001; day 30 vs day 60: p = 0.009.

Type of visit	OSDI score, mean ± SD	Change from baseline, mean ± SD
Baseline	14.62 ± 9.73	
Day 15	11.07 ± 8.41	-3.55 ± 6.89
Day 30	9.50 ± 8.06	-5.12 ± 7.32
Day 60	7.35 ± 6.80	-7.27 ± 7.19

**Table 3:** The change in ocular surface disease index scores from baseline according to severity of meibomian gland loss score at baseline and time between presentation and first session of IPL, Maharashtra, India The p-values are from the Kruskal-Wallis Test followed by Dunn's test with Bonferroni correction. IPL: intense pulsed light, SD: standard deviation.

Baseline parameters	Change in OSDI from baseline at day 15			Change in OSDI from baseline at day 30			Change in OSDI from baseline at day 60		
Severity of meibomian gland loss score	Mean ± SD	Kruskal-Wallis H statistic	p-value	Mean ± SD	Kruskal-Wallis H statistic	p-value	Mean ± SD	Kruskal-Wallis H statistic	P value
0 to 1	-3.75 ± 6.39	2.32	0.31	-5.23 ± 8.20	0.43	0.81	-7.13 ± 6.53	0.03	0.98
2	-3.06 ± 7.44			-4.96 ± 6.98			-7.47 ± 7.48		
≥3	-4.78 ± 5.95			-5.39 ± 6.73			-6.87 ± 7.65		
Time between presentation and first session of IPL (days)	Mean ± SD	Kruskal-Wallis H Statistic	p-value	Mean ± SD	Kruskal-Wallis H Statistic	p-value	Mean ± SD	Kruskal-Wallis H Statistic	p-value
≤7	-4.23 ± 7.67	3.44	0.18	-4.82 ± 7.57	2.36	0.31	-6.69 ± 7.41	3.44	0.18
8-15	-3.83 ± 6.41			-5.87 ± 7.14			-8.59 ± 6.67		
≥16	-2.43 ± 6.26			-4.76 ± 7.22			-6.72 ± 7.33		

In multivariate mixed-effects models, we found a significant reduction in the OSDI score at every follow-up visit (estimate: −2.34, 95% CI: −2.60, −2.07, p < 0.001). Additionally, the mean OSDI scores were lower with a per-year increase in the age of patients (estimate: −0.24, 95% CI: −0.38, −0.09; p = 0.001). None of the other factors were significantly associated with changes in OSDI in our study (Table 4).

**Table 4:** The estimates and their 95% confidence intervals from mixed effects model to evaluate the ocular surface disease index scores in 200 eyes from 100 patients, Maharashtra, India Akaike Information Criteria: 4922.3, Bayesian Information Criteria: 4992.569. The reference for co-morbidities is "No" and the estimates are for "Yes". IPL: intense pulsed light.

Parameters	Estimate (95% confidence intervals)	p-value
Visit	-2.34 (-2.60, -2.07)	<0.001
Age	-0.24 (-0.38, -0.09)	0.001
Gender		
Female	Reference	
Male	0.23 (=2.51, 2.98)	0.87
Comorbidities		
Diabetes mellitus	-0.78 (-2.96, 1.41)	0.49
Hypertension	-0.69 (-3.05, 1.68)	0.57
Glaucoma	2.69 (-2.02, 7.41)	0.26
Lipid disorders	-2.02 (-5.74, 1.70)	0.29
Others	1.11 (-2.67, 4.90)	0.56
Severity of meibomian gland loss at baseline scores		
0 to 1	Reference	
2	-0.02 (-1.19, 1.14)	0.97
≥3	0.06 (-1.61, 1.72)	0.95
Time between the presentation and the first session of IPL		
≤7 days	Reference	
8 to 15 days	0.69 (-2.74, 4.13)	0.69
≥16 days	1.11 (-2.23, 4.45)	0.51

The procedure was well tolerated, and none of the patients reported any side effects (such as redness, other inflammation, or any periocular burns).

## Discussion

We found a significant reduction in OSDI scores at the last follow-up visit compared with the values before the first session of IPL. There was also a significant reduction in OSDI scores after each session. Additionally, there was a significant increase in the proportion of eyes with higher lipid layer thickness after three IPL and MGX sessions in patients with MGD. At the last follow-up visit, a higher proportion of eyes showed improvement in lipid layer thickness. The IPL sessions were well tolerated, with no reported side effects.

Although IPL was initially used in dermatological conditions, its use in ophthalmology has also been well documented [34]. Multiple authors have published reports on the utility of IPL in MGD with or without other therapies. For instance, Wei and colleagues found a significant decrease in OSDI scores in MGD patients after treatment with IPL and meibomian gland expression [68]. However, their mean baseline OSDI scores were higher than those in our study population, indicating a more severe form of dry eye disease. Another study by Chen and colleagues assessed the change in OSDI scores after three sessions of IPL and found a significant reduction in these scores after 120 days compared with baseline values [69]. They also classified the outcome as effective if the OSDI score decreased by 5 points or more and found that effective outcomes were associated with MGD severity but not with baseline OSDI scores [69]. Another study by Gupta and co-workers also found a significant reduction in OSDI scores, but there was no significant difference in the outcomes according to the baseline OSDI scores [70]. Perez-Silguero and coworkers found a reduction in OSDI scores immediately after treatment with IPL and low-level light therapy, but an increase in the scores later, even though they remained significantly lower at the last follow-up [71]. In our study, although there was a higher reduction in OSDI scores in those in whom IPL treatment was started within seven days of diagnosis compared with those who were started after 15 days, it was not statistically significant. Furthermore, at the last visit, the reduction in scores was nearly similar. Thus, there may be an initial larger reduction in OSDI scores in those in whom IPL was started early, but overall, the reduction did not differ. However, age was significantly associated with OSDI scores, and older individuals had lower mean scores overall during the treatment period.

The other important outcome we assessed was the change in lipid layer thickness after IPL therapy. Other authors have also evaluated various aspects of meibomian glands and lipids after treatment with IPL sessions. Ahmed and colleagues found that anionic phospholipids improved after treatment with IPL in patients with MGD [72]. Arita and colleagues compared IPL with MGX with a control group in a randomized clinical trial and found an increase in lipid layer thickness only in the IPL+MGX group [73]. In another multicenter study, Arita and co-workers found that even though the meibum grade and lid margin abnormality scores improved in patients after IPL and MGX treatment, there was no significant improvement in meiboscores [74]. Other authors have also reported improvement in various meibomian gland parameters, such as meibomian glands yielding clear secretion, meibomian glands yielding liquid secretion, and meibomian gland yielding secretion score, but not in meiboscores [75]. Shin and colleagues compared IPL+MGX with IPL alone in a crossover trial and found that meibomian gland expressibility and meibomian quality recovered faster after IPL treatment [76]. In our study, we found that the grade of lipid layer thickness had improved significantly at the last follow-up visit; however, it was not associated with the percentage of meibomian gland loss at baseline or the time of IPL after presentation and diagnosis of MGD. Overall, a higher proportion of eyes showed improvement in the grade of lipid layer thickness. Furthermore, none of the patients reported any side effects after IPL sessions.

Limitations

The study had limitations. This was a retrospective study with limited follow-up of patients and no control group. This was an analysis of clinical data and not a controlled experimental setting. The diagnosis of dry eye and MGD was based on clinical signs and symptoms, and the IPL sessions, along with MGX, were initiated after treatment was offered to these patients. Thus, some patients were started within the first week of presentation, while others started later. However, most patients received their first IPL session within the first month of presentation. There may not have been severe or refractory cases, as indicated by the OSDI scores at baseline. However, all patients had meibomitis. Previous studies have evaluated the effect in more severe forms and refractory cases [73,77]. Nonetheless, our findings suggest that IPL+MGX may improve outcomes in milder cases as well. We evaluated two outcome measures: OSDI scores (for dryness) and changes in lipid layer thickness. Other studies assessed parameters such as inflammatory markers or meibomian gland parameters [35,72,78,79]. We used mixed-effects models, which are suitable for correlated data (as we had both eyes from the same patient) and adjusted for potential confounders, thus providing an added advantage to our analyses. Thus, the results have to be understood within the context of these limitations.

## Conclusions

In this real-world experience scenario, we found that three sessions of IPL along with meibomian gland expression significantly reduced OSDI values after each session. This reduction was maintained even after 30 days from the last IPL session. The age of the patient was also significantly associated with OSDI scores; older individuals had lower OSDI scores over the follow-up period. Additionally, we observed an increase in lipid layer thickness over time following IPL sessions. The severity of meibomian gland loss and the duration between presentation and the first session were not significantly associated with changes in OSDI scores or changes in lipid layer thickness in these patients. None of the patients reported any side effects after the procedure. Therefore, IPL along with meibomian gland expression may be offered as a treatment modality even for milder forms of dry eye symptoms associated with meibomian gland dysfunction.
